# Dampened Transient Actuation of Hydrogels Autonomously Controlled by pH-Responsive Bicontinuous Nanospheres

**DOI:** 10.1021/acsami.4c02643

**Published:** 2024-04-03

**Authors:** Wouter P. van den Akker, Rolf A. T. M. van Benthem, Ilja K. Voets, Jan C. M. van Hest

**Affiliations:** † Department of Chemistry & Chemical Engineering, Institute for Complex Molecular Systems, Bio-Organic Chemistry, Eindhoven University of Technology, Helix, P.O. Box 513, 5600MB Eindhoven, The Netherlands; ‡ Department of Chemistry & Chemical Engineering, Self-Organizing Soft Matter, Eindhoven University of Technology, P.O. Box 513, 5600MB Eindhoven, The Netherlands; § Department of Chemistry & Chemical Engineering, Laboratory of Physical Chemistry, Eindhoven University of Technology, P.O. Box 513, 5600MB Eindhoven, The Netherlands; ∥ Shell Energy Transition Center Amsterdam, Grasweg 31, 1031 HW Amsterdam, The Netherlands

**Keywords:** soft actuators, bicontinuous nanospheres, urease, transient behavior, permeability

## Abstract

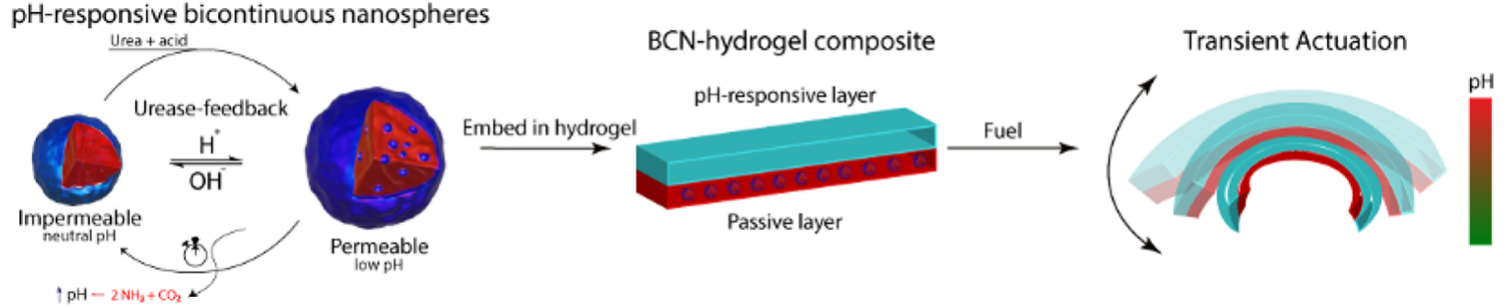

The fabrication of a soft actuator with a dampened actuation response is presented. This was achieved via the incorporation into an actuating hydrogel of urease-loaded pH-responsive bicontinuous nanospheres (BCNs), whose membrane was able to regulate the permeability and thus conversion of fuel urea into ammonia. The dampened response of these nanoreactors to the enzymatically induced pH change was translated to a pH-responsive soft actuator. In hydrogels composed of a pH-responsive and nonresponsive layer, the transient pH gradient yielded an asymmetric swelling behavior, which induced a bending response. The transient actuation profile could be controlled by varying the external fuel concentrations. Furthermore, we showed that the spatial organization of the BCNs within the actuator had a great influence on the actuation response. Embedding the urease-loaded nanoreactors within the active, pH-responsive layer resulted in a reduced response due to local substrate conversion in comparison to embedding them within the passive layer of the bilayer hydrogel. Finally, we were able to induce transient actuation in a hydrogel comprising two identical active layers by the immobilization of the BCNs within one specific layer. Upon addition of urea, a local pH gradient was generated, which caused accelerated swelling in the BCN layer and transient bending of the device before the pH gradient was attenuated over time.

## 1 Introduction

Stimuli-responsive materials are capable of responding to an (external) environmental cue, such as pH, light, or temperature, and have found widespread use in applications such as drug delivery,^1^ (bio)sensing,^2^ and actuation.^3^ Traditionally, stimuli-responsive materials can be switched between two states, for which both a trigger and counter trigger are required. This is in contrast to a new class of adaptive materials that are able to autonomously adjust their properties without the need for an external counter stimulus to revert back to their original configuration.^4−6^

Multiple examples of these transient materials have been reported in recent years. Klajn and co-workers developed self-erasing inks based on the reversible aggregation of photoactive nanoparticles bearing azobenzene moieties.^7^ Aizenberg et al. showed the development of microstructured systems capable of pH-responsive chemo–mechanical–chemo cycles as well as a chemo–thermomechanical feedback loop.^8^ Transient materials have also been realized utilizing ATP (and its hydrolysis) in ATP-assembling systems,^9−12^ DNA strand displacement reactions,^13−15^ and more broadly applicable dissipative chemical reaction networks, which typically activate a precursor molecule into an active product that participates in the self-assembly of various materials.^16−19^ For example, Walther et al. showed the development of a transient mechanical actuation device based on a one-component pH-feedback system using self-decarboxylating tribromoacetic acid, which was responsible for transient pH flips.^20^

A highly versatile manner to construct transient materials is through the incorporation of (enzymatic) feedback loops, which are able to sense environmental changes and trigger a response. These often require external substrates, and the lifetime of the specific response is readily programmed by variation of the substrate or catalyst concentration, gaining temporal control over system functionality. A common method to achieve such adaptivity is by incorporation of pH-feedback systems.^6,21^ This way, the dissipative conditions of these pH-regulatory feedback loops are able to drive stimuli-responsive materials out of equilibrium.^19^

Multiple enzymes can be utilized in these pH-feedback systems, such as urease, esterase, and glucose oxidase.^22^ Moreover, in pH-feedback systems the complexity can be increased by the introduction of an antagonistic enzyme to elicit a (programmable) countertrigger.^23−26^ Urease in particular has been used extensively in the fabrication of transient materials, such as the assembly of pH-responsive polymers,^27,28^ autonomous “breathing” of microgels,^29^ or the transient gelation of hydrogels^30^ because of its pH-dependent bell-shaped activity profile, which enables the enzyme to show nonlinear behavior. This specific feature has also been used to construct pH-responsive nanoreactors whose membrane permeability was regulated by the urea–urease feedback loop.^23,31−33^

We previously reported on the self-regulating properties of urease-loaded pH-responsive nanoreactors, based on polymersomes and porous bicontinuous nanospheres (BCNs).^33^ The latter showed unique self-regulating properties, as a temporal membrane collapse was observed due to locally produced ammonia inside the hydrophilic pores, leading to dampened catalytic behavior. We were interested in investigating if the BCNs’ self-regulating features could be transferred to macroscopic systems by integrating these nanoreactors into an actuating material. A suitable matrix material for this purpose is hydrogels. The hydrogel matrix allows the facile inclusion of the nanoreactors in a mechanically robust material without hampering the (rapid) diffusion of solutes and exchange of reactants and products. An additional advantage of hydrogels is their ease of fabrication and modification. Functional groups, such as pH-responsive or temperature-responsive moieties, can be incorporated with ease to achieve controlled swelling and deswelling, which could lead to mechanical actuation.

Here, we report on the construction of an actuating material in which urease-loaded BCNs were incorporated. While enzymatic or feedback loop driven actuation has been shown previously,^34,35^ we show that the actuation can be regulated by the physical integration of our nanoreactors and that the spatial organization of these nanoreactors plays a pivotal role in the actuation response. We first demonstrated that the BCN nanoreactors retained their catalytic self-regulating profile when embedded in the hydrogel. Furthermore, by constructing a hydrogel comprising a pH-responsive and nonresponsive layer transient actuation was attained. This actuation response was highly dependent on fuel concentrations and indeed showed dampening behavior, demonstrating that the features of the nanoreactors could be transferred to the macroscopic material. The positioning of the BCNs in the hydrogel determined the level of response that could be achieved, as immobilization of the nanoreactors in the pH-responsive active layer resulted in a diminished actuation response in comparison to immobilization in the passive layer. Finally, we demonstrate that actuation can also be achieved in a bilayer hydrogel with an identical composition of each layer. By spatially controlling the positioning of the BCNs in one of the two identical layers, the induced pH gradient caused accelerated swelling in the respective BCN layer and therefore temporal bending until the pH gradient was attenuated by diffusion, causing the actuator to attain its original shape.

## 2 Experimental Section

### 2.1 Materials

All materials were used as received, unless stated otherwise.

Horseradish peroxidase from *Amoracia rusticana* (Type VI, 295 U/mg) and urease from *Canavalia ensiformis* (Type IX, 72.5 U/mg) were purchased from Sigma-Aldrich. 2-(Diethylamino)ethyl methacrylate (99%), poly(ethylene glycol) methyl ether 2-bromoisobutyrate (*M*_w_/*M*_n_ 1.07, mPEG macroinitiator), poly(ethylene glycol) methacrylate (*M*_n_ 360), 2-hydroxyethyl methacrylate (98%), acrylic acid (99%), acrylamide (99%), ethylene glycol dimethacrylate, urea (≥98%), 2,2′-azinobis(3-ethylbenzothiazoline-6-sulfonic acid) diammonium salt (98%), urea, lithium phenyl-2,4,6-trimethylbenzoylphosphinate (≥95%), and Rhodamine B isothiocyanate were purchased from Sigma-Aldrich. 4-Methacryloyloxy benzophenone was purchased from TCI Chemicals. Alexa Fluor 647 NHS ester (99%) was purchased from ThermoFischer. 2-(Diethylamino)ethyl methacrylate was passed through an alumina column to remove inhibitor.

### 2.2 Synthesis of Amphiphilic Poly[ethylene glycol]-*b*-Poly[diethylaminoethyl Methacrylate-*g*-Benzophenone Methacrylate] MPEG_45_-*b*-P[DEAEMA_175_-*g*-BMA_28_]

Note that the same polymer is used in this study as in our previously published procedure.^33^

mPEG- macroinitiator (0.05 mmol, 100 mg), diethylaminoethyl methacrylate (DEAEMA) (10 mmol, 2 mL) and benzophenone methacrylate (BMA) (1.12 mmol, 300 mg) were added to a Schlenk flask and dissolved in 6 mL of 2-butanone. Subsequently, 2,2′-bipyridine (0.1 mmol, ∼16 mg) was added to the solution. The CuBr (0.1 mmol) catalyst was added, and the flask was immediately frozen in liquid N_2_ and subjected to three freeze–pump–thaw cycles before placing it in a preheated oil bath at 60 °C. After the reaction, the solution was diluted with 60 mL of THF and passed through an alumina column to remove the catalyst. The filtrate was concentrated and precipitated in cold hexane to give the final polymer.

### 2.3 Formation of Bicontinuous Nanospheres

The labeling of urease with Rhodamine B (RhB) and the labeling of horseradish peroxidase (HRP) with AlexaFluor647 (AF647) were done according to previously published procedures.^33^ 15 mg of (RhB-)urease and/or 2 mg of (AF647-)HRP were dissolved in 1 mL of 5 mM phosphate buffer (pH 7.4) in a 4 mL vial equipped with a magnetic stirrer rotating at 500 rpm. The block copolymer mPEG_45_-*b*-p[DEAEMA_175_-*g*-BMA_28_] was dissolved in THF to obtain a final concentration of 5 mg/mL. 1 mL of polymer solution was added to 1 mL of enzyme solution with a flow rate of 1 mL/h via a syringe pump. The cloudy suspension was immediately cross-linked by UV irradiation and transferred to a 12–14 kDa MWCO membrane and dialyzed against 5 mM phosphate buffer (pH 7.4), while occasionally refreshing the phosphate buffer. Next, the formed nanoreactors were transferred to a 1 MDa Float-A-Lyzer and dialyzed against 5 mM phosphate buffer overnight to remove nonencapsulated enzymes. Finally, the nanoparticles were centrifuged for 5 min at 4000 rpm via spin filtration over 0.1 μm membranes. The filtrate was analyzed for dye absorbance to check whether any nonencapsulated enzymes were still present. In that case, the nanoreactors were redispersed in 1 mL of 5 mM phosphate and subjected to another spin filtration cycle.

### 2.4 Photo-Cross-Linking of Bicontinuous Nanospheres

For nanoreactor cross-linking, the formed nanoreactors were placed in a UV photoreactor and irradiated (365 nm) for 5 min at room temperature with a light intensity of 5 mW cm^–2^.

### 2.5 Synthesis of Hydroxyethyl Methacrylate–Poly(ethylene glycol) Methacrylate–Ethylene Glycol Dimethacrylate Hydrogel

400 μL of 2-hydroxyethyl methacrylate, 300 μL of 5 mg/mL BCN solution, 80 μL of PEG360 methacrylate, 1 μL of ethylene glycol dimethacrylate as cross-linker, and 1 mg/mL LAP photoinitiator were thoroughly mixed and sonicated for 1 min. The monomer mixture was irradiated with UVA (365 nm) light with an intensity of 5 mW cm^–2^ for 5 min. In typical experiments, 100 μL of this monomer mixture was added to an individual well of a 96-well plate and subsequently photopolymerized. For illustrative purposes, the monomer mixture was polymerized in a 4 mL vial to show hydrogel formation.

### 2.6 Formation of BCN-Loaded Hydrogel for Enzymatic Assays

Prior to formation of the hydrogels, the amount of encapsulated enzyme was determined by UV/vis spectroscopy, and samples were diluted to obtain enzyme concentrations of 40 U/mL for urease and 25 U/mL for HRP. A typical assembly with only urease yields 5 mg/mL nanoreactors with ∼120 U/mL encapsulated urease. Subsequently, 300 μL of enzyme-loaded BCN solution was mixed with 400 μL of 2-hydroxyethyl methacrylate, 80 μL of PEG360-methacrylate, 1 μL of ethylene glycol dimethacrylate, and 1 mg/mL LAP photoinitiator and thoroughly mixed. 100 μL of this solution was dispersed per well in a 96-well plate, and the plate was irradiated with UVA (365 nm) light with an intensity of 5 mW cm^–2^ for 5 min to form the BCN-loaded hydrogels.

### 2.7 pH Evolution of Urease-Loaded BCNs vs Free Urease within the Hydrogel Matrix

For the pH evolution of the urease-loaded BCNs, 50 μL of substrate solution was added to each individual well of a 96-well plate. This substrate solution consisted of 50 mM urea and 0.03 mg/mL C-SNARF-4F ratiometric pH dye in 5 mM phosphate buffer at pH 5. The final substrate concentrations for the experiment were 16.6 mM urea and 0.01 mg/mL C-SNARF-4F. The C-SNARF-4F ratiometric dye was excited at λ = 525 nm, and the emission at λ = 587 and 650 nm was measured.

### 2.8 HRP-Urease-Loaded BCNs and Distinct Populations

For the HRP-urease-loaded BCN experiments presented in Figure 2C,D, 300 μL of HRP-urease-loaded BCN solution was mixed with 400 μL of 2-hydroxyethyl methacrylate, 80 μL of PEG360-methacrylate, 1 μL of ethylene glycol dimethacrylate, and 1 mg/mL LAP photoinitiator and thoroughly mixed. 100 μL of this solution was dispersed per well in a 96-well plate, and the plate was irradiated with UVA (365 nm) light with an intensity of 5 mW cm^–2^ for 5 min to form the BCN-loaded hydrogels.

50 μL of substrate solution was added to each individual well. This substrate solution consisted of 50 mM urea, 20 mM ABTS, 10 mM H_2_O_2_, and 5 mM phosphate buffer at pH 5. The final substrate concentrations for the experiments were 16.6 mM urea, 6.6 mM ABTS, and 3.3 mM H_2_O_2_. The absorbance at λ = 415 nm was monitored over time.

For the experiment in which two distinct BCN populations were employed, separate batches of BCNs were assembled composed of HRP-loaded BCNs and urease-loaded BCNs. These were compared with one population in which both enzymes were coencapsulated. Samples of all populations were diluted to obtain similar enzyme concentrations prior to the experiments shown in Figure 2C,D. UV/vis spectra of urease-HRP BCNs, AF647-HRP BCNs, and RhB-urease BCNs are found in Figure S3.

### 2.9 Synthesis of Bilayer Hydrogel (AAc:AAm/AAm)

The bilayer hydrogel was manufactured by partially photocuring (2.5 min, 5 mW cm^–2^) 400 μL of an initial monomer mixture (10 wt % acrylamide (AAm), 50:1 monomer:cross-linker with *N*,*N*′-methylenebis(acrylamide) (MBA) as cross-linker) in a poly(lactic acid) (PLA) mold (30 × 5 × 6 mm^3^). Subsequently, 400 μL of the second monomer mixture (10 wt % AAm, 0.5 wt % acrylic acid (AAc), 50:1 AAm:MBA) was added on top, and the system was photocured for another 6 min. The hydrogel was then removed from the mold and equilibrated in buffer for at least 24 h to properly swell. 0.01 mg/mL fluorescein acrylate was copolymerized into the passive layer for visibility. The photoinitiator that was used was lithium phenyl-2,4,6-trimethylbenzoylphosphinate (LAP). The BCNs were mixed within either the passive acrylamide layer or the active acrylic acid layer depending on the type of experiment.

For the double active bilayer hydrogel, both layers consisted of 5% AAc (0.5 wt % AAc–10 wt % AAm, 50:1 AAm:MBA). In this instance, BCNs were embedded within one of the two active layers. First, the active layer without the BCNs was polymerized for 2.5 min, after which the BCN layer was added and polymerized for another total of 6 min. Urease concentrations in the actuation experiments were 120 U/mL, in which the BCN volume was 400 μL for the fabrication of a specific layer.

### 2.10 Actuation Experiments

The synthesized bilayer hydrogels (as described in Section 2.6) were equilibrated in 50 mL of 1 mM phosphate buffer or 50 mL of 1 mM citrate buffer for the experiment described in Figure 4.

Normal actuation experiments (1 mM phosphate, pH 4.5) were started upon the addition of urea. Transient actuation experiments (equilibrated at pH 7.4) were started upon acidification of the medium to pH 4.5 and the addition of urea (10, 50, or 200 mM).

Photos were made every 2 or 5 min using a smartphone camera (Open Camera app), and data were analyzed using ImageJ to obtain bending angles.

## 3 Results and Discussion

**Figure 1:**
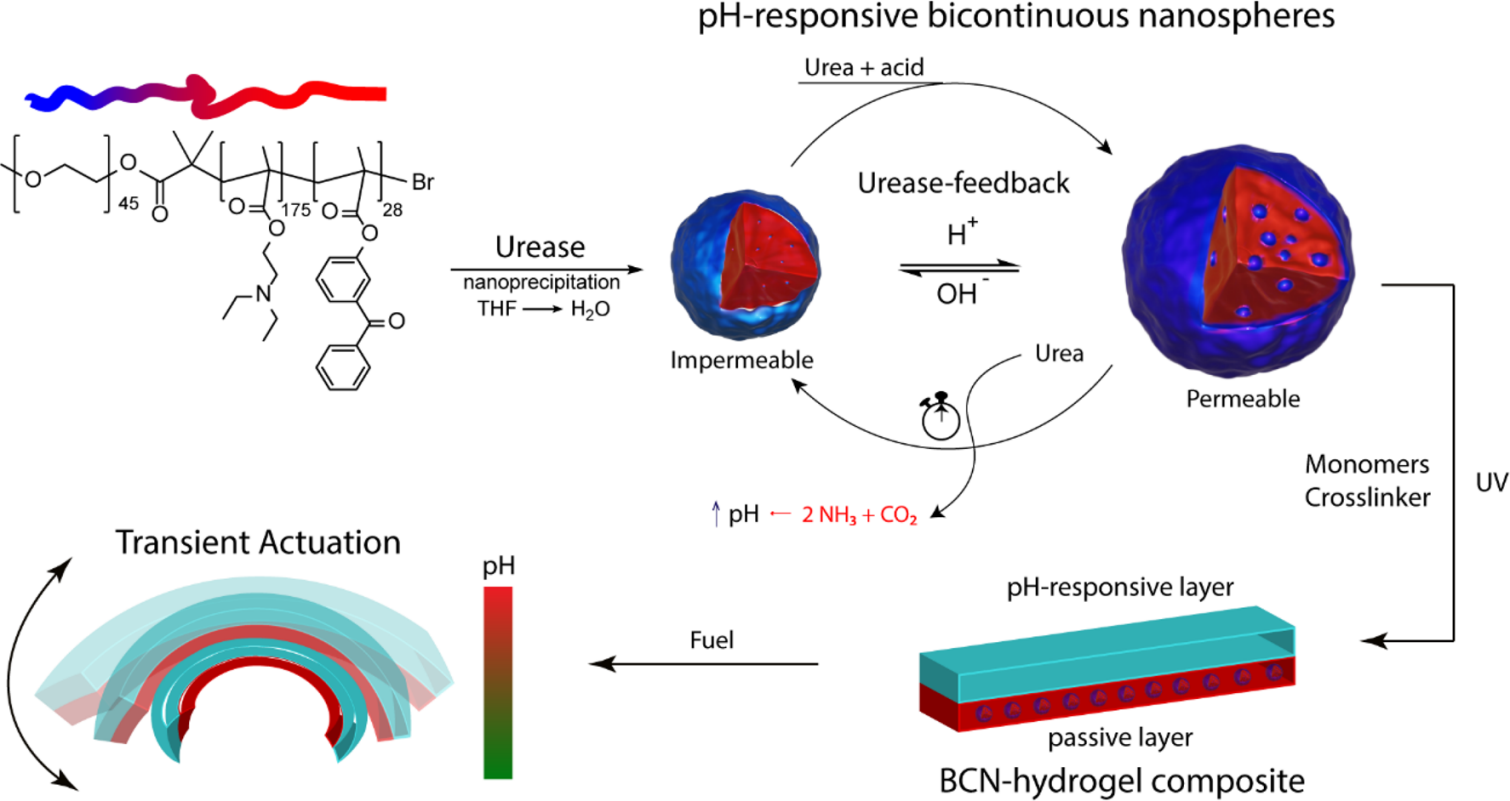
Schematic illustration of transient actuation controlled by pH-responsive bicontinuous nanospheres (BCN). At neutral pH, the BCN is impermeable for the substrate. Upon acidification, substrate urea can be converted into product ammonia, which results in a pH increase, creating a feedback loop with dampening features. This permeability switch is utilized to autonomously regulate a soft actuation device based on a pH-responsive (bilayer) hydrogel.

The design of our dampened actuating device is depicted in Figure 1. As matrix material, a bilayer hydrogel was used, of which at least one of the two parts was pH-responsive (swelling at high pH), via the incorporation of acrylic acid moieties. As actuating units, we constructed the bicontinuous nanospheres (BCNs) following our earlier reported procedures.^33^ In short, the polymer building blocks were prepared by ATRP and were composed of a hydrophilic poly(ethylene glycol) block and a hydrophobic domain comprising pH-responsive 2-(diethylamino)ethyl methacrylate (DEAEMA) and 4-(methacryloyloxy)benzophenone (BMA) as monomers. To steer the assembly into the BCN morphology, the block copolymer was designed to have a significant hydrophobic fraction, and its final composition was mPEG45-*b*-p[DEAEMA175-*g*-BMA28]. The polymer was self-assembled by the nanoprecipitation method, and the enzymes horseradish peroxidase (HRP) and urease were encapsulated by dissolving them in the aqueous solution prior to self-assembly. The formed suspension was then irradiated by UV light in order to cross-link the interior of the nanoreactor due to the radicals generated by the benzophenone methacrylate. This cross-linking prevented dissociation of the assembly at lower pH, but instead caused significant swelling accompanied by the permeability switch upon acidification. Previous analysis by dynamic light scattering (DLS) and cryo-TEM indicated a pH-responsive size increase from 250 to 400 nm by decreasing the pH from 8 to 5, which was reversible for at least 5 cycles. The previously published characterization (DLS, cryo-TEM) of these BCNs is summarized in Figure S1.^33^

The BCNs were immobilized first into a nonresponsive hydrogel by dispersing the particles in a pregel monomer solution prior to the photopolymerization using lithium phenyl-2,4,6-trimethylbenzoylphosphinate (LAP) as photoinitiator. This monomer solution consisted of hydroxyethyl methacrylate (HEMA), PEG350 methacrylate (PEGMA), and ethylene glycol dimethacrylate as cross-linker (EGDMA) in a monomer ratio of 625:50:1, respectively.

**Figure 2:**
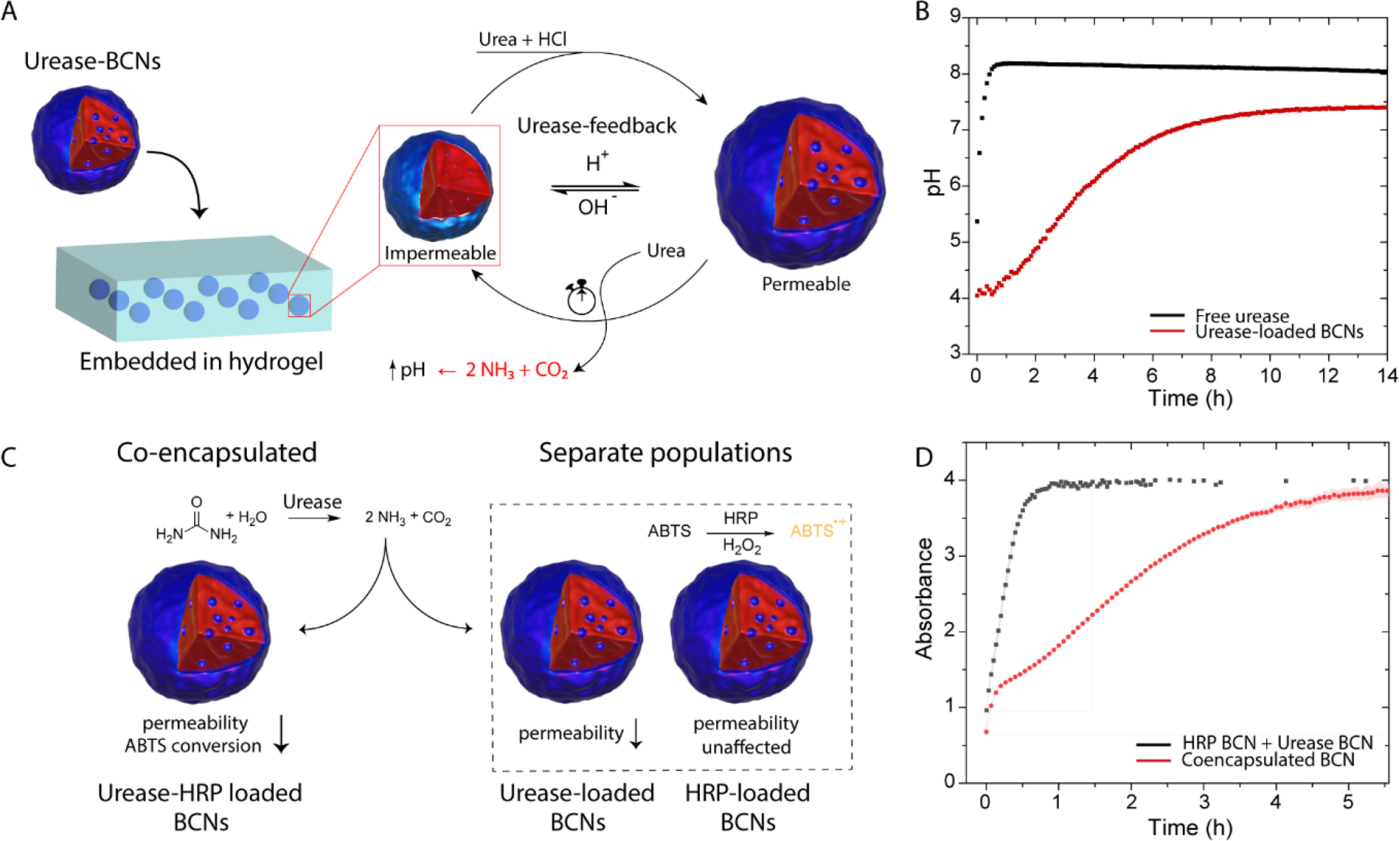
Characterization of the BCN nanoreactor performance when embedded within a hydrogel matrix. (A) Schematic representation of the feedback loop. The urea–urease feedback loop autonomously regulates the membrane permeability of the BCNs. Once sufficient urea is converted into ammonia to increase the pH, the BCNs become impermeable for substrate. (B) pH evolution of urease-loaded BCNs versus free urease—both embedded within the hydrogel matrix, showing the dampened effect caused by the membrane feedback of the BCNs. (C) Separation of enzymes (HRP and urease) in distinct populations removes the previously described nonlinear effect, as there is no local ammonia production to influence the HRP-loaded BCN population. (D) Determination of HRP-catalyzed ABTS^•+^ formation by the co-encapsulated BCNs and the separate populations by measuring the absorbance at λ = 415 nm. Experimental conditions: 40 U/mL urease, 25 U/mL HRP, 5 mM phosphate buffer, 16.6 mM urea, 6.6 mM ABTS, and 3.3 mM H_2_O_2_.

We then compared urease-loaded BCNs embedded within the hydrogel with immobilized urease and monitored the pH evolution over time using a ratiometric dye named C-SNARF-4F, enabling a real-time fluorescent readout by comparing 587/650 nm emissions (Figure S2). When nonencapsulated urease was immobilized within the gel, there was no membrane inhibiting its substrate influx, and the pH increased continuously until pH ∼8.2, which corresponds to the upper detection limit of the dye.^36^ After the experiment, the pH was validated using a pH meter and was measured to be ∼9.2 for the immobilized urease, which corresponds to the p*K*_a_ of ammonium. For urease-loaded BCNs, the pH-responsive permeability switch provided a negative feedback loop, which caused the urea influx to be gradually more inhibited as the pH increased until the nanoreactors were no longer permeable for the substrate. Indeed, Figure 2B showed that for urease-loaded BCNs, the pH stagnated at pH ∼7.4, corresponding to the pH at which the nanoreactors became impermeable. Hence, we have shown that we are able to translate the earlier reported urease feedback loop into a hydrogel matrix, with as the main difference that the response times were increased as a result of the hydrogel environment.

After the functionality of the feedback loop was established, we moved on to loading the nanoreactors with both urease and a second enzyme, horseradish peroxidase (HRP), which displayed a relatively pH-independent activity profile. As such, the HRP activity was mainly controlled by the permeability of the BCN, which was regulated by the local production of ammonia by urease. The synthesis of ABTS^•+^ from ABTS by HRP (Scheme S3) enabled us to follow the permeability profile of the nanoreactors. After an initial unhindered conversion of ABTS to ABTS^•+^ a “dampening phase” was observed, during which a temporal reduction in output was measured. This dampening phase originated from the local ammonia produced by urease, leading to a high local pH, causing the BCNs to become temporarily less permeable for both urea and ABTS, after which a steady-state phase was obtained. This effect observed for BCNs embedded in the hydrogel (Figure 2D, red curve) was similar to our previously reported free BCN nanoreactors.^33^

To further verify this nonlinear behavior was not caused by the hydrogel diffusional barrier, we repeated the experiment with two separate populations. One BCN population consisted of urease-loaded BCNs and the other of HRP-loaded BCNs (Figure 2C,D), while total enzyme concentrations remained similar. In this instance, the locally produced ammonia of the urease-BCN population was unable to directly influence the membrane permeability of the HRP-loaded BCNs, and we therefore did not expect any nonlinear phase to occur. Indeed, separation of the enzymes in distinct populations resulted in rapid production of ABTS^•+^ with no decrease in catalytic output until maximum absorbance was reached, in a remarkably shorter time span in comparison to the co-encapsulated condition. This emphasized the influence of the locally produced ammonia in the HRP-urease-loaded nanoreactors. When the urease-HRP BCNs were equilibrated at pH 8 before addition of ABTS, negligible substrate conversion was observed (Figure S4). These experiments confirmed that the urease feedback loop and the catalytic profile of the BCNs were both maintained within the hydrogel environment.

**Figure 3:**
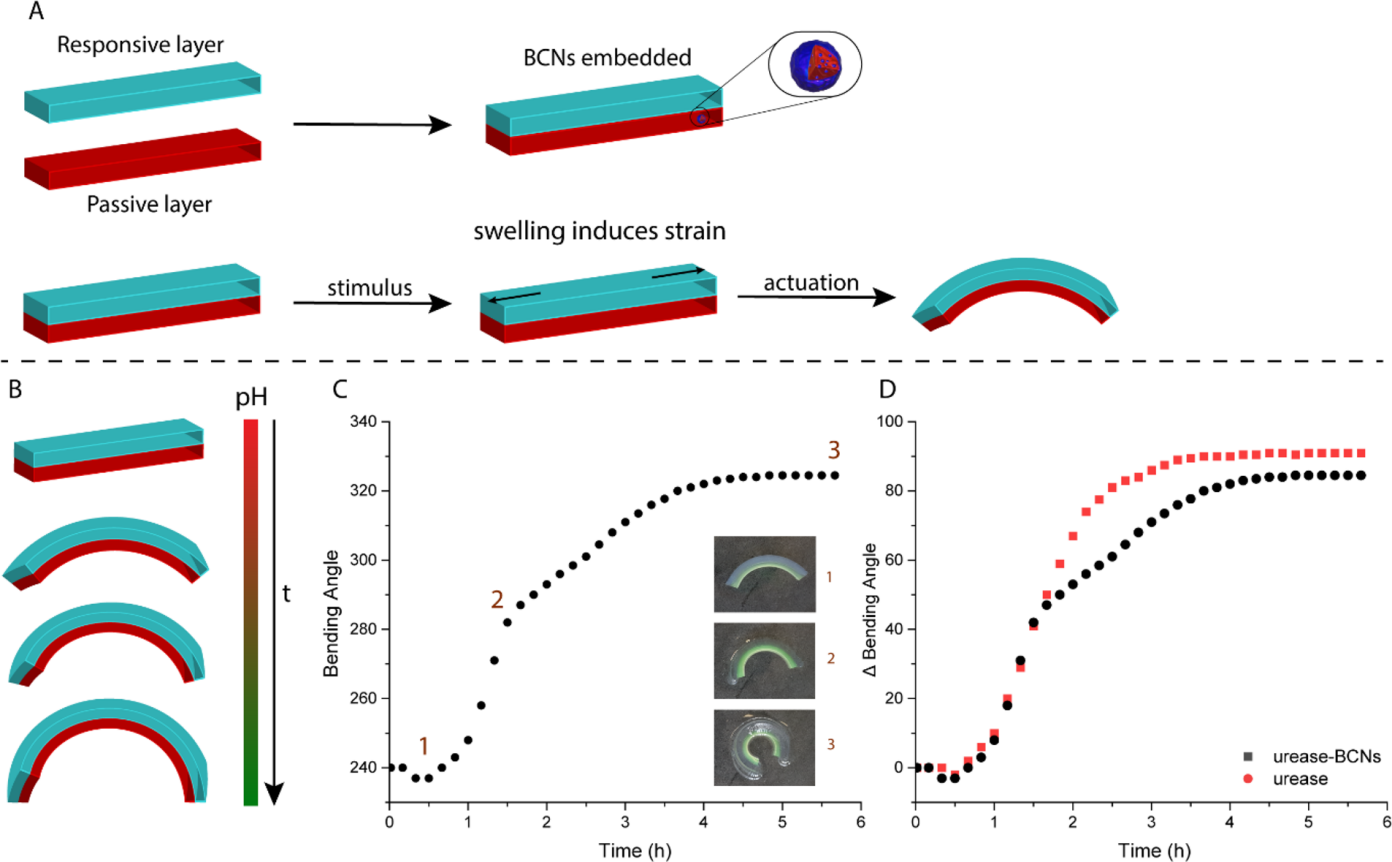
(A) Design of bilayer hydrogel soft actuators. The responsive layer contains acrylic acid (AAc) and acrylamide (AAm) and *N*,*N*′-methylenebis(acrylamide) (MBA) as cross-linker. The passive layer consists of AAm and MBA (50:1 AAm:MBA, 10 wt % AAm). Upon swelling or shrinking of the responsive layer, strain is induced, which subsequently causes bending. (B) Conversion of urea into ammonia by the urease-loaded BCNs leads to an increase in pH over time, causing the actuator to bend. (C) Bending profile, and corresponding snapshots, of a 5% AAc bilayer hydrogel with urease-loaded BCNs embedded in the passive layer. (D) Actuation comparison between urease-loaded BCNs and free urease in the hydrogel. Experimental conditions: 120 U/mL of urease (BCN layer has a volume of 400 μL), 1 mM phosphate buffer, and 200 mM urea.

Next, we set out to investigate whether BCNs were able to regulate a soft actuator. For this purpose, a bilayer hydrogel was fabricated, which is a common strategy to introduce a mechanical response toward physicochemical stimuli.^37−39^ Typically, the passive layer consists of monomers that do not respond toward external stimuli, such as acrylamide, whereas the active, responsive layer is made of a monomer capable of responding to the stimulus of choice (i.e., temperature, light, pH). The responsive layer then swells/shrinks under the influence of the external stimulus, which causes the bilayer hydrogel to bend as the passive layer does not undergo morphological changes. In our system, the pH-responsive layer was composed of acrylic acid(AAc) mixed with acrylamide (AAm), and the passive, nonresponsive layer was made of 10 wt % AAm. This creates anisotropy, so that the system is strained upon swelling/shrinking of the active layer (Figure 3A). The bilayer hydrogel was manufactured by partially photocuring (2.5 min, 5 mW cm^–2^) 400 μL of an initial monomer mixture in a PLA mold (30 × 5 × 6 mm^3^) and subsequently adding 400 μL of the second monomer mixture, after which the system was photocured for another 6 min. For visibility purposes, fluorescein acrylate was copolymerized into the passive layer. The hydrogels were equilibrated in buffer for at least 24 h in order to achieve full hydration prior to the bending experiments.

First, it was investigated whether the BCNs were able to autonomously regulate the actuator. The urease-loaded BCNs were embedded within the passive layer of the bilayer hydrogel by mixing it with the monomer solution prior to the photopolymerization and equilibrated at pH 4.5, and upon the addition of urea, the bending angle over time was monitored. As the pH increased upon the conversion of urea into ammonia, the acrylic acid moieties were deprotonated which resulted in significant swelling, causing the bilayer hydrogel to bend to a considerable extent, as illustrated in Figure 3C. We defined a “straight” gel to have a bending angle of 180° and a fully bent gel (with AAc as outer layer) to have a bending angle of 360°. Gratifyingly, the hydrogel bending also displayed nonlinear dampening behavior. As is clear from the inclination point depicted as 2 in Figure 3C, after an initial sharp bending process, this was attenuated in the second phase of the actuation. To demonstrate that this was the result of the confinement of urease in the BCNs, we also performed the same experiment with free urease embedded in the passive layer of the hydrogel. Indeed, Figure 3D shows that in the initial stages urease and urease-loaded BCNs behaved in a similar fashion. However, at approximately *t* = 2 h, the free urease actuation continued to progress at a similar rate until it reached its plateau at a somewhat higher Δ bending angle (8°). The effect of pH on the 1D-swelling ratio and bending angle of the bilayer hydrogels is shown in Figure S5.

Control experiments showed that without AAc in the active layer, no actuation was observed. While the BCNs were pH-responsive due to their diethylamine moieties, the swelling of the nanoreactors themselves was insufficient to cause bending in the macroscopic hydrogel (Figure S7).

**Figure 4:**
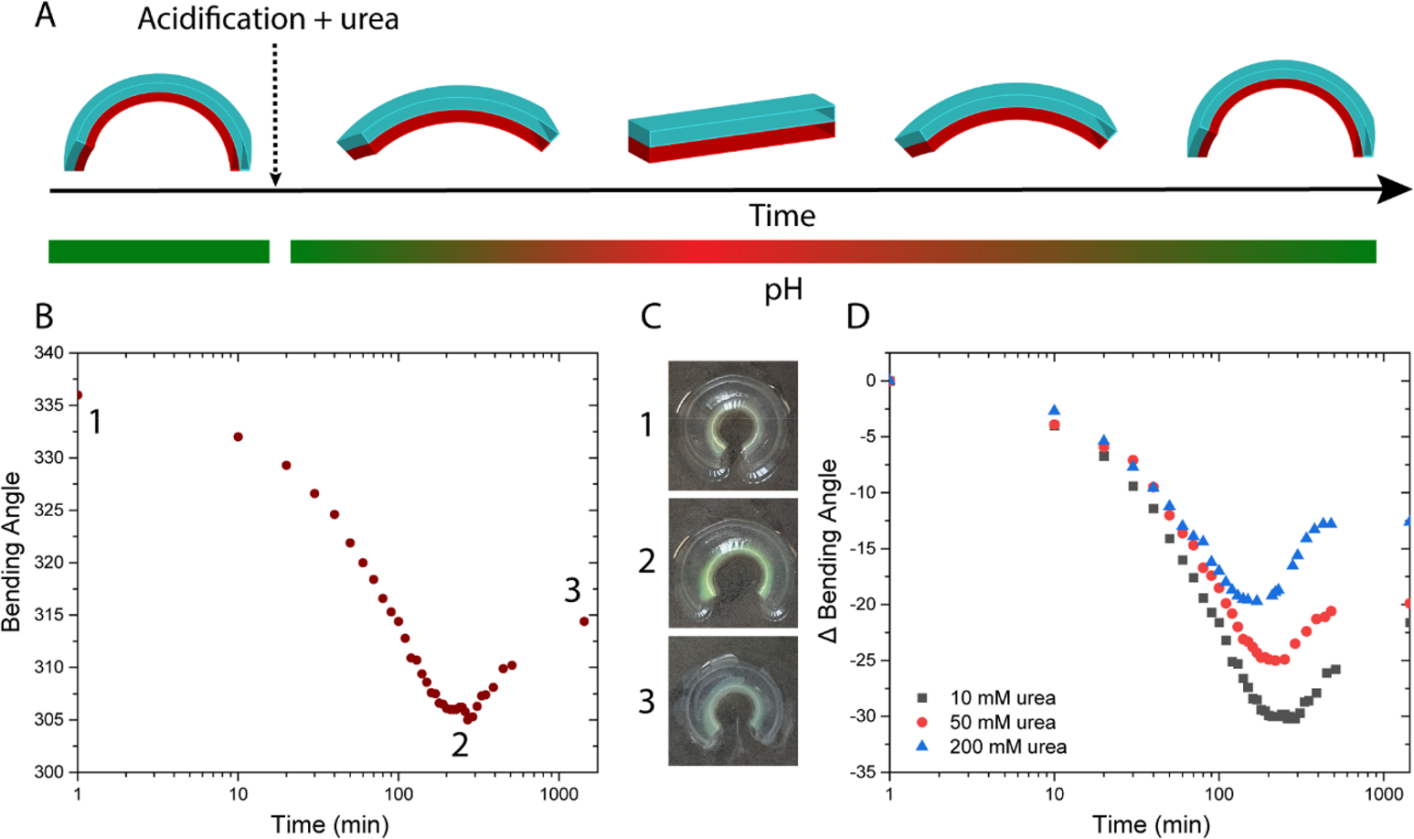
(A) Transient actuation of bilayer hydrogels. Upon acidification and addition of urea, the actuator initially starts unbending because of protonation of the carboxylates in the responsive layer prior to bending due to the conversion of urea into basic ammonia. (B) Transient actuation of a 5% AAc bilayer hydrogel using 10 mM urea. (C) Snapshots of the soft actuator at specific time points shown in part B. (D) Influence of various urea concentrations on the transient actuation profile. Experimental conditions: 120 U/mL urease.

After establishing the conditions under which our BCNs demonstrated dampened behavior in the hydrogels, we investigated the transient actuation of our device by the simultaneous addition of both acid and urea. The BCNs were embedded in the passive layer of our actuator and equilibrated in 1 mM buffer, pH 7.4, prior to the actuation experiment, in order to reach an at-equilibrium bent state. Note that at this pH the BCNs are in a less permeable state. The solution was then acidified to pH 4.5 with simultaneous urea addition, and the bending angle was measured over time. In this way, the actuator gradually started unbending due to the drop in pH and the diffusional influx of protons into the BCN-loaded layer, causing them to switch to their more permeable state, while simultaneously urea was converted into ammonia, which led to a pH increase as feedback. We initially investigated various compositions with different acrylic acid contents and found that 5% AAc (respective to AAm fraction) was most effective for these actuator dimensions, whose actuation profile and corresponding snapshots are shown in Figure 4B,C. Lowering the AAc content to 1% resulted in ineffective actuation, as the initial bending angle was significantly lower and the range of deformation of the hydrogel was limited (Figure S8). The 10% AAc composition performed quite similarly to 5% AAc. However, at 10% AAc the actuator was fully bent at pH 7.4 (360°).

We then varied the urea concentrations and investigated their influence on the transient actuation profile. In this instance, citrate buffer (1 mM) was used as its lower p*K*_a_ caused larger differentiation between urea concentrations. Low urea concentrations such as 10 mM resulted in an increased range of initial (acid driven) deformation in comparison to 200 mM urea (Figure 4D), as ammonia was produced at a slower rate in the former case while the rebending response (ammonia driven) was similar in terms of bending angle difference. Compared to actuation started at acidic conditions, we observed a smaller actuation window due to the competition of two processes, both limited by (“slow”) mass transport. The actuator responds to the drop in pH, which protonates the poly(acrylic acid) side chains causing the swollen responsive layer to deswell and unbend. At the same time the BCNs become permeable, and the substrate is converted to ammonia, gradually increasing the pH which causes the actuator to bend again. These competing processes, however, do allow for careful tuning of the actuation behavior simply by varying the external fuel concentrations. This transient actuation cycle could be restarted by acidification and the addition of urea (10 mM); however, a reduction in actuation potential and speed was observed, presumably due to the accumulation of NH_3_/NH_4_^+^ (Figure S11).

**Figure 5:**
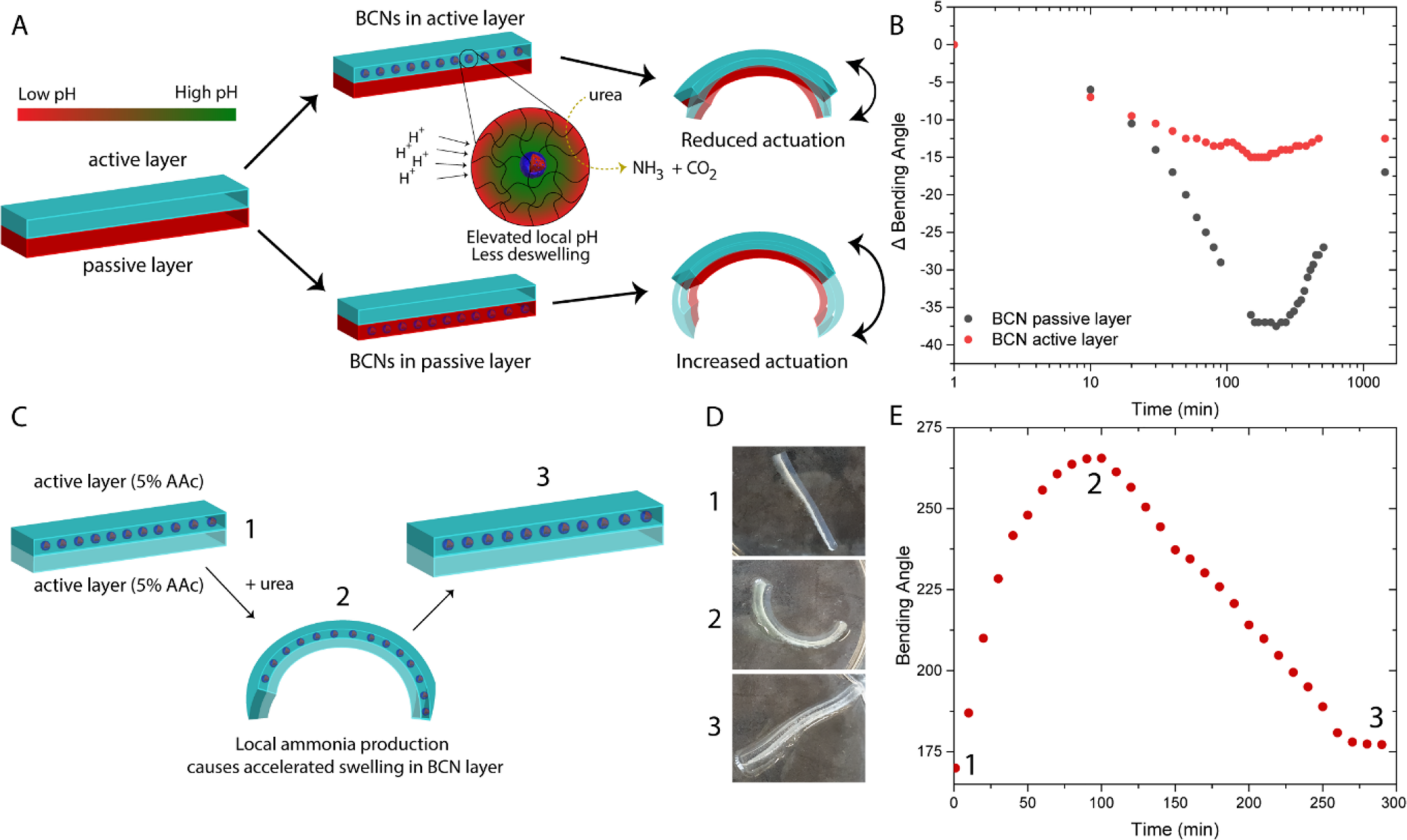
(A, B) Difference in transient actuation amplitude by embedding the BCNs in the active layer instead of the passive layer. When BCNs are embedded in the active layer, higher local pH diminishes the actuation potential (5% AAc actuator). The actuator is equilibrated at pH 7.4. (C) Schematic representation of actuation in a double-pH-responsive hydrogel by means of a pH gradient. Local ammonia produced in the BCN layer causes accelerated swelling in that respective layer, until the gradient is no longer maintained. (D) Corresponding snapshots of 5%AAc-BCNs/5%AAc hydrogels. (E) Bending angle over time in the double-pH-responsive hydrogel. Time points correspond to snapshots shown in 5D. Experimental conditions: actuator is equilibrated in 50 mL of 1 mM phosphate buffer, 200 mM urea, and 120 U/mL urease.

Next, we compared embedding the BCNs in the passive layer with embedding them in the active layer. We envisioned that embedding the nanoreactors in the active, pH-responsive layer would result in a decrease in actuation, as the urea conversion, and therefore base production, takes place in proximity of the acrylic acid moieties and, as such, is able to compete with the acidification of the medium more directly and, hence, more effectively, as illustrated in Figure 5A. In this way, the spatial organization of our catalytic nanoreactors would directly influence the macroscopic response of the actuator, as the local pH in the responsive layer is higher. In the case of embedding the BCNs in the passive layer, the base would still need to diffuse toward the responsive, acrylic acid layer. Indeed, by embedding the BCNs in the active layer, we observed a significantly diminished actuation response, as illustrated in Figure 5B. Once again, the actuators were equilibrated at pH 7.4 and actuated by acidification toward pH 4.5 while simultaneously adding urea. While embedding the BCNs within the passive layer resulted in a transient actuation response of ∼40°, embedding the BCNs in the active layer diminished the response to ∼15°, emphasizing the importance of spatial organization. Furthermore, the pH evolution of both systems progressed in a similar fashion (Figure S10), ruling out a large difference in the level of ammonia production over time.

Finally, we investigated whether we could utilize this local pH effect to induce bending in a fully pH-responsive actuating device. For this purpose, the hydrogel was now composed of two identical layers comprising 5% AAc, with the BCNs embedded within one of those two active layers, as illustrated in Figure 5C. When the actuator was equilibrated at pH 4.5, the bending angle was close to 180° (snapshot 1, Figure 5D), confirming that the degree of swelling in both layers was uniform, despite the presence of BCNs in a “swollen” or more permeable state in one of them. However, once urea was added to the system, we observed rapid bending due to the accelerated swelling of the BCN layer due to local conversion of urea into ammonia (snapshot 2). After reaching its maximum bending angle at 100 min, the actuating response gradually diminished as the other active layer also started to swell. After approximately 5 h the actuator returned to its original configuration with a bending angle of 180°, albeit more swollen (snapshot 3; video of double active hydrogel is available in the Supporting Information, Video SV1). Thus, we have shown that with the simple addition of urea we are able to achieve transient actuation induced by a pH-gradient generated within the nanoreactor layer. We envision that such an approach could be utilized to obtain increased system complexity and could be extended toward the use of antagonistic enzymes to provide oscillating actuation.

## 4 Conclusion

In conclusion, we have presented hydrogel actuators with a dampened response through the incorporation of autonomously regulated pH-responsive nanoreactors. The nanoreactors were based on urease-loaded bicontinuous nanospheres (BCNs) that were embedded in the hydrogel matrix material. The membrane-regulated urease feedback loop in these BCNs was still functional within a hydrogel matrix, as demonstrated by their nonlinear dampened catalytic profile. When the BCNs were placed in a bilayer hydrogel actuator comprising a pH-responsive, active layer and a nonresponsive, passive layer, the hydrogel showed responsiveness to the fuel urea as evidenced by pH-induced bending behavior. A clear difference was observed with encapsulated urease within the BCNs, and urease freely loaded in the hydrogel, leading to dampened bending behavior of the actuating device in the former case. The simultaneous addition of both an acidic stimulus and fuel resulted in transient deformation of the actuator, whose response could be modulated by varying fuel concentrations.

Furthermore, we investigated whether the spatial organization of the BCNs influences the actuation response. When the BCNs were dispersed in the passive layer, a more distinct response was observed than when the BCNs were embedded within the active layer. The diminished actuation response of the latter system was caused by the more direct effect of the higher local pH due to the local ammonia production within the respective layer. Finally, we utilized this local ammonia production to induce bending in an actuator comprising two identical active layers in which the BCNs were embedded within only one layer. Upon the addition of urea, rapid transient bending was observed due to accelerated swelling of the BCN layer, indicating that we were able to induce complex bending behavior by generation of local pH gradients.

## Supporting Information Available

Instruments used and supplementary schemes and figures (PDF)
Video SV1: transient actuation of a bilayer hydrogel comprising two pH-responsive layers by addition of urea (MP4)
