# Supplementary material for: Dampened Transient Actuation of Hydrogels Autonomously Controlled by pH-Responsive Bicontinuous Nanospheres
Source: ACS Appl Mater Interfaces. 2024 Apr 3;16(15):19642–50. doi: 10.1021/acsami.4c02643 (PMC11040580; doi:10.1021/acsami.4c02643)
Supplement: Supplementary file 1 — am4c02643_si_001.pdf [file am4c02643_si_001.pdf]

Supporting Information

Dampened transient actuation of hydrogels autonomously controlled by pH-responsive bicontinuous nanospheres

Wouter P. van den Akker[1,2], Rolf A.T.M van Benthem[3,4], Ilja K. Voets[2], Jan C.M. van Hest[1*]

[1]Department of Chemistry & Chemical Engineering, Institute for Complex Molecular Systems, Bio-Organic Chemistry, Eindhoven University of Technology, Helix, P.O. Box 513, 5600MB Eindhoven (The Netherlands)

[2]Department of Chemistry & Chemical Engineering, Self-Organizing Soft Matter
Eindhoven University of Technology, P.O. Box 513, 5600MB Eindhoven (The Netherlands)

[3]Department of Chemistry & Chemical Engineering, Laboratory of Physical Chemistry. Eindhoven University of Technology, 5600MB Eindhoven (The Netherlands)

[4]Shell Energy Transition Center Amsterdam
Grasweg 31, 1031 HW Amsterdam (The Netherlands)

Corresponding Author
j.c.m.vanhest@tue.nl

1. Instruments

Nuclear Magnetic Resonance Spectroscopy (NMR). ^1H NMR spectra were recorded on a Bruker (400MHz) spectrometer with $CDCl_3$ as solvent.

Gel-Permeation Chromatography (GPC). The dispersity of the polymer was measured using a Shimadzu Prominence-I SEC system with a PL gel 5 µm mixed D and mixed C column (Polymer Laboratories) calibrated with PS standards and equipped with a Shimadzu RID-20A differential refractive index detector. THF was used as an eluent with a flow rate of 1 mL/min.

Dynamic Light Scattering (DLS). DLS measurements were performed on a Malvern Zetasizer Nano ZSP at room temperature.

Plate reader. Time-dependent absorbance and fluorescence readings were measured on a Tecan™ Spark 10M plate reader using Corning Falcon 96 Black Flat Transparent well plates. Tecan™ Spark 10M has linear OD measurements between 0-4 OD.

UV Photoreactor. The crosslinking of the nanoreactors was performed using a Luzchem **LZC-4V** photoreactor equipped with 14 UVA lamps with a wavelength of 365 nm and power density of 5 mW/cm^2.

2. Supplementary figures

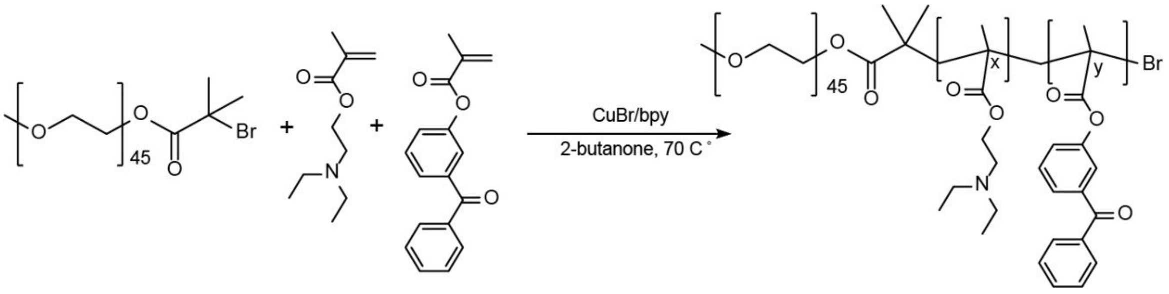

Scheme S1. Synthesis of mPEG-b-p[DEAEMA-g-BMA] by ATRP

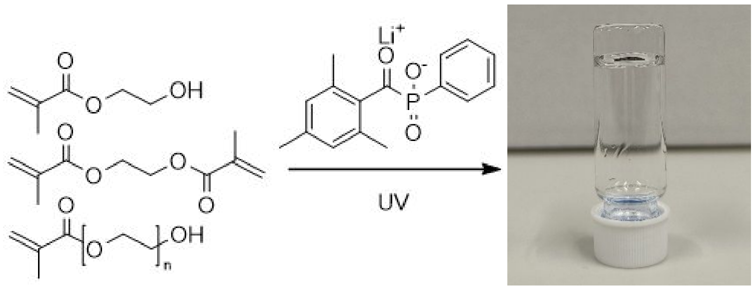

Scheme S2. Synthesis of HEMA-PEG360MA-EGDMA based hydrogel

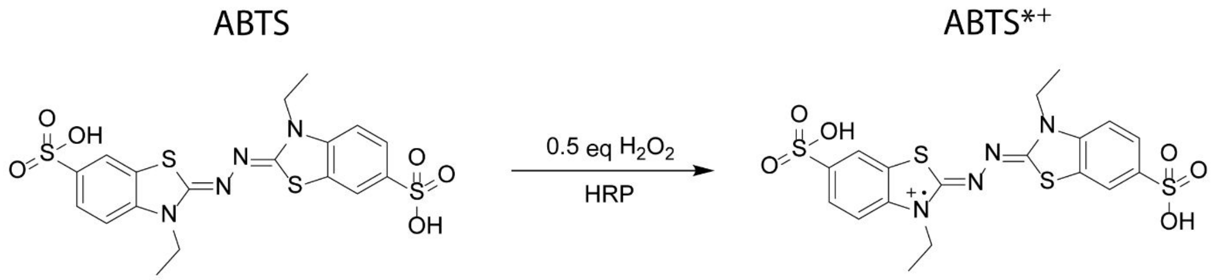

Scheme S3: Enzymatic conversion of 2,2′-azino-bis(3-ethylbenzothiazoline-6-sulfonic acid) to ABTS^{*+} by horse radish peroxidase.

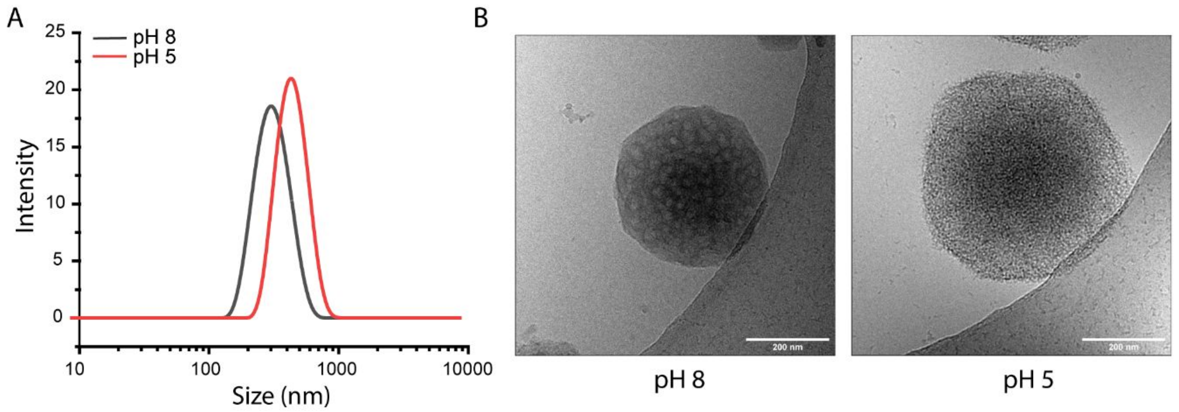

Figure S1. A) DLS analysis of BCNs at pH 5 and pH 8. B) Cryo-TEM analysis of BCNs. Scalebar represents 200 nm. Note that this data is summarized from reference 1, as the same BCNs are used.

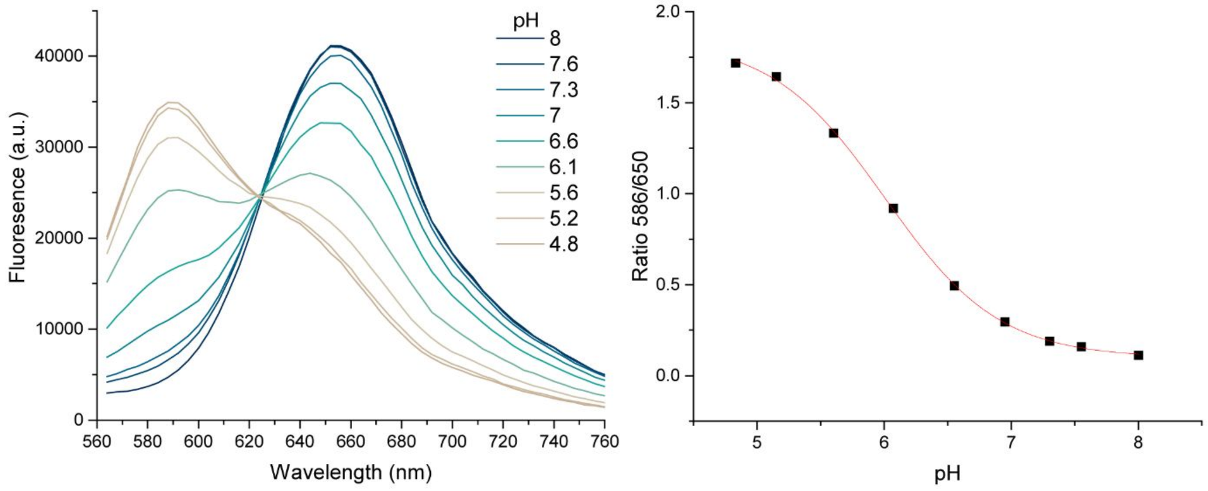

Figure S2. C-SNARF-4F ratiometric pH sensor emission spectrum & calibration for pH measurements. Note that this dye was also previously used in ref 1.

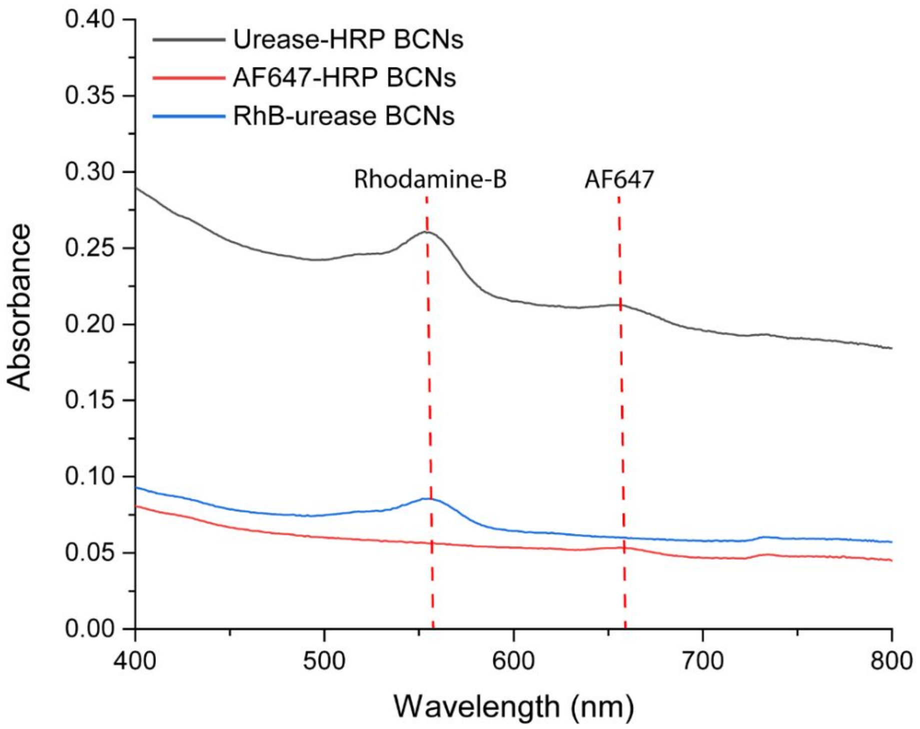

Figure S3. UV-Vis spectra of urease-loaded BCNs, HRP-loaded BCNs and urease-HRP loaded BCNs. Urease was labeled with Rhodamine B while HRP was labeled with AF647.

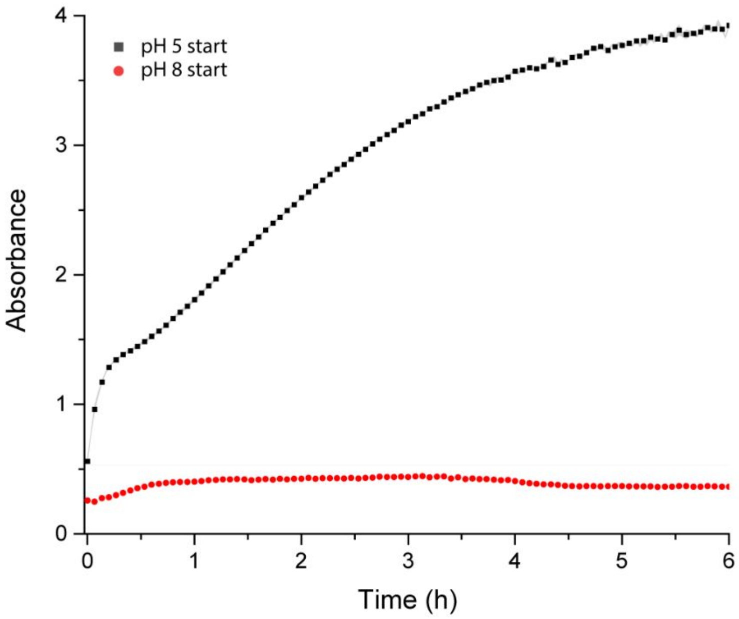

Figure S4. Absorbance-time plot of urease-HRP loaded BCNs at pH 5 and pH 8 starting conditions. (embedded in the hydrogel). Absorbance is measured at 415 nm corresponding to the λ_{max} of ABTS$^{\bullet+}$.

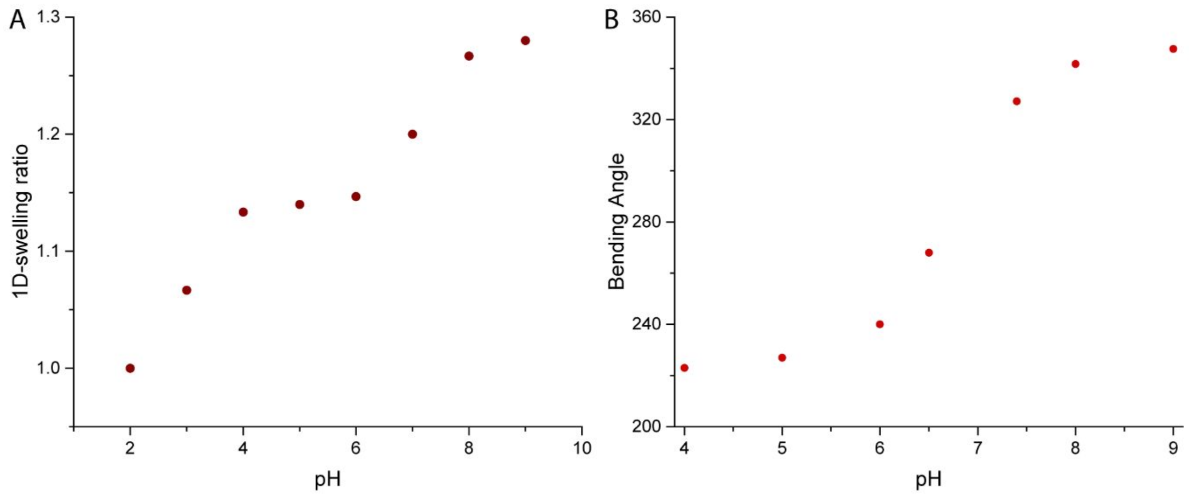

Figure S5. Effect of pH on A) 1D-swelling ratio of 5% AAc hydrogels vs the initial mold dimensions (30x5x6 mm). B) Bending angles of 5% AAc bilayer hydrogels.

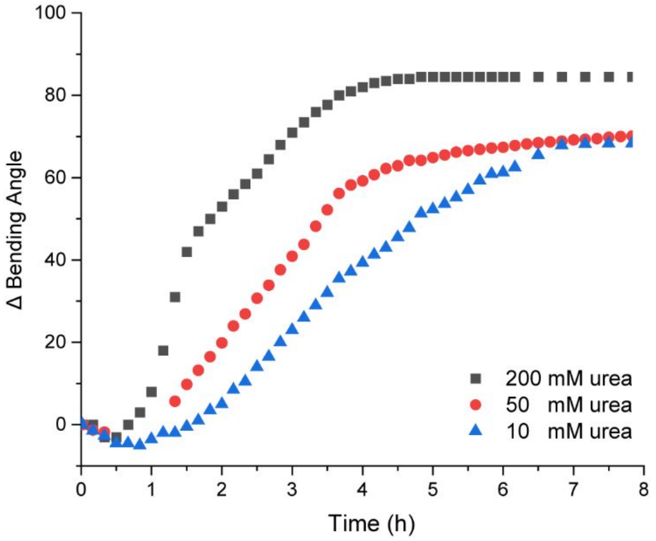

Figure S6. Influence of urea on actuation speed of 5% AAc urease-BCN hydrogels. Hydrogel is composed of a passive AAm layer (10 wt%) and an active layer consisting of AAc and AAm (0.5wt% AAc:10wt%AAm). Urea concentrations vary from 10 mM urea to 200 mM urea.

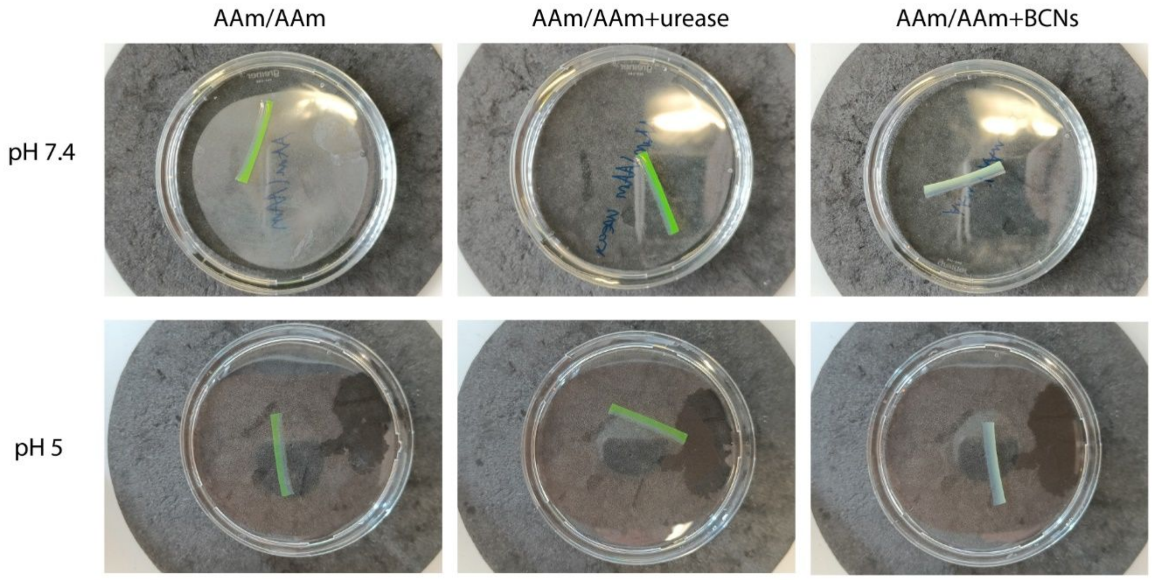

Figure S7. Additional controls. Hydrogel bilayers composed of AAm/AAm layers do not induce bending. Additionally, while the BCNs are pH-responsive, immobilization of the BCNs in AAm/AAm hydrogels does not induce bending at different pH's, indicating the importance of the AAc layer.

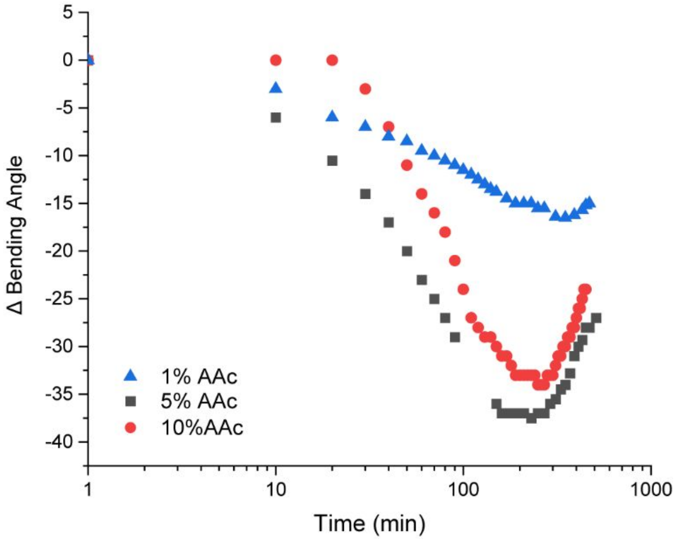

Figure S8. Influence of acrylic acid content on the transient actuation efficiency. Acrylic acid content was varied from 1%, 5% to 10% acrylic acid, respective to the amount of acrylamide.

MBA was used as a crosslinker and AAm as secondary component (50:1 AAm:MBA ratio). In the case of 10% acrylic acid, the final composition was 10 wt% of AAm and 1 wt% of AAc.

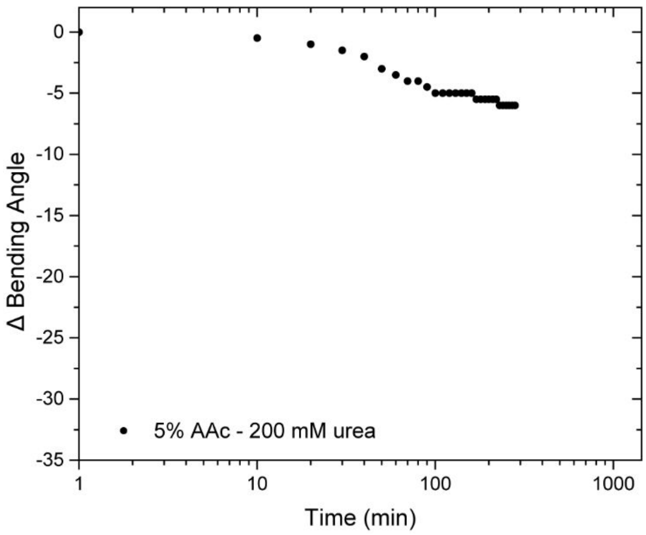

Figure S9. Addition of urea to a 5%AAc actuator equilibrated in 1 mM phosphate buffer, pH 7.4, 200 mM urea causes minor unbending.

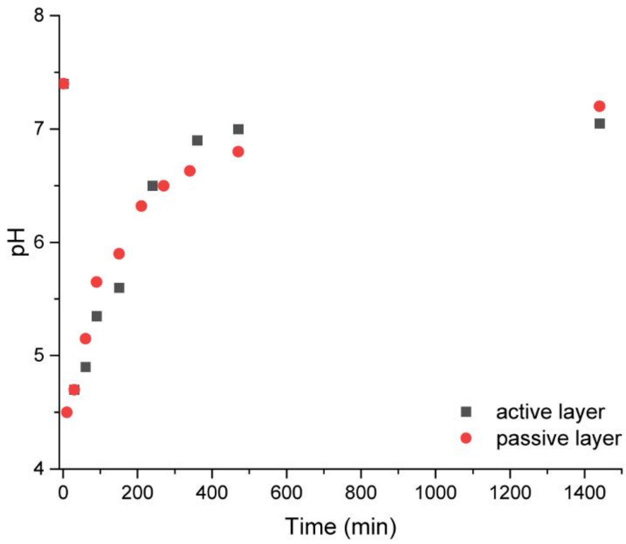

Figure S10. pH evolution of urease-BCN actuators when BCNs are embedded in the active layer (black) and passive layer (red). 5% AAc actuator was used in both instances. The pH evolution corresponds to the bending angle profile shown in Figure 5B.

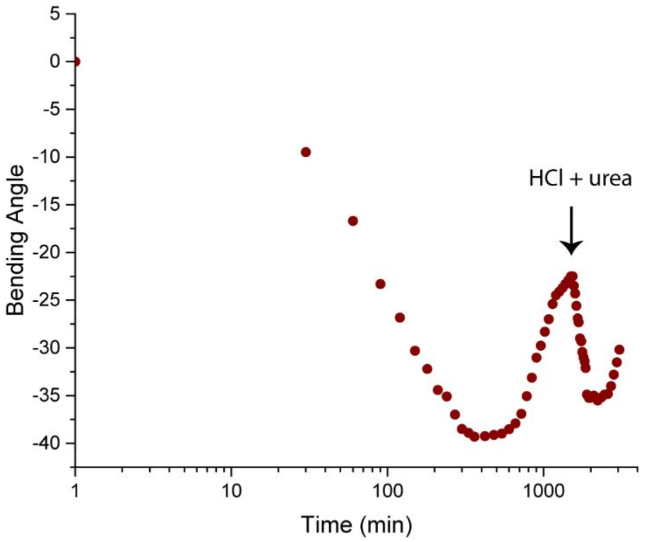

Figure S11. Cyclability of the actuator in a transient actuating experiment. The cycle is restarted by acidification with HCl to pH 4.5 and a supplementary addition of urea (10 mM). The 2nd cycle was slower and had a lower actuation potential compared to the first cycle, as a consequence of the accumulation of NH_3/NH_4^+.
